# 

**Published:** 1889-04

**Authors:** 


### BOOK NOTICES.

THE BANNER OF LIGHT, Boston, Colby and Rich, publishers, has just entered upon its 65th volume. For upwards of thirty-two years this able exponent of the spiritualistic philosophy has kept the field steadily, progressively and unflinchingly in the face of every opposition, supporting its affirmations by an array of facts convincing and satisfactory to the unbiased mind. Luther Colby, the veteran editor-in-chief of the *Banner*, has, from first to last, kept his place at the helm in all weathers, and directed its cou-se of safety alike in calm and in storm, till now his name has become a household word wherever the seed he has sown with so much care and ability have found rooting. May his ripe old age be full of blessings and the *Banner* long of life.

THE PSYCHIC LIFE OF MICRO-ORGANISMS.—A study in Experimental Psychology, by Alfred Binet. Translated from the French by Thomas McCormack, with a preface by the author written especially for the American Edition. Chicago, 1889. The Open Court Publishing Company. Cloth, 75 cents. Paper, 50 cents.

The classification and arrangement of the infinitesimal world of living organisms into distinct families and groups, showing their peculiar forms, instincts and modes of life, is the development of a new science having a most important bearing upon health, since there is no escaping their contact even in the air we breathe, the food we eat and the water we drink. The work has great merit and we commend it for its completeness, not only in a scientific point of view, but likewise as conveying a class of fnformation which only the patient and painstaking investigator in fields beyond the ordinary ken, is able to acquire and impart.

AMERICAN MEDICAL ASSOCIATION.—The fortieth annual meeting of this body will be held at Newport, R. I., commencing June 25th, and continue for four days, during which the usual themes in medicine and surgery will be presented and discussed. A large attendance is anticipated.

BABYLAND FOR BABIES AND OUR LITTLE MEN AND WOMEN, for tender youths, are precisely what is claimed for them, the very best juvenile publications anywhere to be had. D. Lathrop & Co., Boston Publishers.

THE CENTURY MAGAZINE, never runs dry of the very essence of good things. The April number is uncommonly rich. We don't quite see how people can manage to do without it. Century Co., New York City.

Our acknowledgements are due for the following publications :

OPHTHALMOLOGY AND OTOLOGY, by Seth S. Bishop M. D. Chicago.

ELECTROLYSIS in certain cases, by Robert Newman M. D., Philadelphia.

ELEVENTH ANNUAL REPORT, of the Presbyterian Eye Ear, and Throat Charity Hospital, Baltimore.

COMMERCIAL TRAVELER.—How to be successful on the road. Fowler & Wells Co., New York.

FOOD VERSUS BACILLI, IN CONSUMPTION. By Ephraim Cutter, M. D. S. L. D., New York City.

NASAL AND NEUROTIC FACTORS, etc., reprint from New York Medical Journal.

YELLOW FEVER, protection by Scientific Quarantine. By Wolfred Nelson C. M. M. D. New York City.

CATARRHAL INFLAMMATIONS, by Ely McClennon M. D., Chicago.

THE NEURAL AND PSYCHO-NEURBL FACTOR ETC. By C. H. Hughs M. D. St. Louis.

